# An Architecture Providing Depolarization Ratio Capability for a Multi-Wavelength Raman Lidar: Implementation and First Measurements

**DOI:** 10.3390/s17122957

**Published:** 2017-12-20

**Authors:** Alejandro Rodríguez-Gómez, Michaël Sicard, María-José Granados-Muñoz, Enis Ben Chahed, Constantino Muñoz-Porcar, Rubén Barragán, Adolfo Comerón, Francesc Rocadenbosch, Eric Vidal

**Affiliations:** 1 CommSensLab, Department of Signal Theory and Communications, Universitat Politècnica de Catalunya (BarcelonaTech-UPC), 08034 Barcelona, Spain; msicard@tsc.upc.edu (M.S.); maria.jose.granados@tsc.upc.edu (M.-J.G.-M.); enis.benchahed@gmail.com (E.B.C.); constan@tsc.upc.edu (C.M.-P.); ruben.barragan@tsc.upc.edu (R.B.); comeron@tsc.upc.edu (A.C.); roca@tsc.upc.edu (F.R.); 2 Space Sciences and Technologies-Research Center for Aeronautics and Space/Catalan Institute for Space Studies (CTE-CRAE/IEEC), BarcelonaTech University (UPC), 08034 Barcelona, Spain; 3 Politecnico di Torino, 10129 Torino, Italy; 4 UTC Fire & Security España SL, 08950 Esplugues de Llobregat, Spain; Eric.Vidal2@fs.utc.com

**Keywords:** lidar system, depolarization channel, calibration, stability, depolarizing particles

## Abstract

A new architecture for the measurement of depolarization produced by atmospheric aerosols with a Raman lidar is presented. The system uses two different telescopes: one for depolarization measurements and another for total-power measurements. The system architecture and principle of operation are described. The first experimental results are also presented, corresponding to a collection of atmospheric conditions over the city of Barcelona.

## 1. Introduction

Multi-wavelength Raman lidars provide a number of measurements that can be used to characterize the nature and origin of the aerosols present in the atmosphere. The most relevant [1] are: the lidar ratio, defined as that between the retrieved extinction and backscattering at a given wavelength; the quotient of lidar ratios at two different wavelengths and the color ratio or the Ängstrom exponent [2], also computed by comparing the retrieved backscattering and extinction at different wavelengths.

Additionally, since the 1970s the use of the lidar depolarization technique has proven to be a valuable tool for atmospheric sciences, see for instance [3] or [4]. Regarding aerosol characterization, the depolarization information has been widely used for aerosol typing when combined with additional optical properties (e.g., [5,6,7,8,9,10]). In this sense, it can also be very useful in the retrieval of the atmospheric boundary layer (ABL) height since it allows to discriminate between the aerosol within this layer and different aerosol types coupled to the ABL height based on aerosol data [11]. In reference [12] it is shown how the depolarization data combined with the color ratio allow for discriminating among different kinds of aerosols and clouds, so depolarization information can be added to the set of parameters to be considered in aerosol classification [12,13].

Besides aerosol typing, the depolarization technique also provides relevant information for the retrieval of aerosol microphysical properties. Due to the particle shape information associated to lidar depolarization, the detection of non-spherical particles can be highly improved (see for instance [14,15,16,17,18]).

Because of the importance of depolarization measurements for aerosol science, a new depolarization measurement channel [19] has been developed and implemented for the BarcelonaTech (UPC) 6-channel elastic/Raman lidar [20]. The majority of the currently working systems [8,21,22,23,24,25,26] use a single telescope and either a polarizing beam-splitter that separate the parallel and perpendicular polarization components of the light collected by the telescope or a non-polarizing beamsplitter in one of whose outputs a polarizer is inserted; these approaches present the issue of needing a very precise characterization of the crosstalk parameters of the beam-splitters. The main innovation of our system is the use of an additional telescope (in fact, a telephoto lens) to measure the cross-polarized return signal (and thus, the un-polarized component of this signal), without altering the original system. The light collected by the main telescope is coupled into the wavelength separation unit using an optical fiber bundle that produces a nearly total effective depolarization at its output, making the rest of the system insensitive to changes in polarization.

The rest of this article is organized as follows: Section 2 describes the system architecture; Section 3 contains the basic formulation for retrieving the information about depolarization profiles; Section 4 details the calibration process and Section 5 presents some measurements corresponding to a collection of atmospheric conditions over the city of Barcelona.

## 2. System Architecture

A complete description of the UPC main lidar instrument can be found at [20]. The transmitter is based on a Quantel^®^ Brilliant^®^ laser (Quantel Laser, Les Ulis, France), equipped with second and third harmonic generators. The laser produces 4-ns pulses at with energies 130 mJ at 1064 nm and 532 nm and 40 mJ at 355 nm. The beam divergence, according to the manufacturer, is less than 0.7 mrad [27].

**Figure 1:**
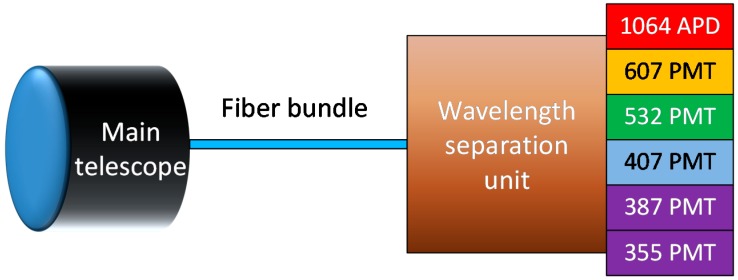
Main receiver of UPC Raman lidar [20], presenting the telescope, the fiber bundle and the wavelength separation unit, which delivers the collected light to the different receivers: an avalanche-photodiode (APD) for 1064 nm and photo-multiplier tubes (PMT) for the other channels.

The 6-channel main receiver unit is sketched in Figure 1. A 356-mm diameter, 3910-mm focal length telescope (C14-A XLT, CELESTRON^®^, Torrance, CA, USA) collects the backscattered light and couples it (with the help of a field lens) to a 3-mm diameter, 3-m long fiber bundle (custom-made, CeramOptec^®^, Bonn, Germany). The estimated field of view of this optical arrangement is approximately 1.2 mrad. The bundle delivers the light to a wavelength separation unit, which splits it to the different channels. They include: three elastic backscattering channels (1064, 532 and 355 nm) and three Raman channels (607 and 387 nm, for nitrogen excited at 532 and 355 nm, respectively, and 407 nm, for water vapor excited at 355 nm).

The axes of the laser beams and the telescope are parallel, with an approximate distance of 30 cm between them. This fact causes the partial overlap between the part of the atmosphere illuminated by the laser beams and that “seen” by the receivers, which affects to the amount of light collected from short distances [28,29,30,31,32,33,34,35].

We have tested the polarization performance of the fiber bundle [36], finding that, for a linearly polarized input, the power values measured at the output of a polarization analyzer show a standard deviation of less than 1%. Therefore we can consider that the light coming out of the fiber bundle shows a nearly total effective depolarization. This fact permits to consider that the 6 channels (including the 532-nm one, implied in the measurements presented in Section 5) are basically sensitive to the total collected power, without any polarization discrimination, even though the wavelength separation unit includes several beam-splitters that could cause di-attenuation. The overall calculated transmission of the fiber bundle and the wavelength separation unit at the 532-nm output is 6.18% [20]. Further measurements (see Section 4) suggest that the 532-nm channel transmission could be lower.

The signal collected by the different channels is detected by means of an avalanche photo-diode (APD) photo-receiver (for the 1064 nm channel) and five photo-multiplier tube modules (PMT) (for the remaining channels) and digitized by a parallel Licel^®^ Transient Recorder [37] (Licel GmbH, Berlin, Germany) with analog and photon-counting capabilities.

**Figure 2:**
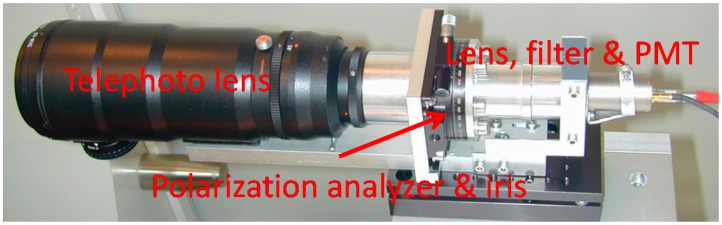
Auxiliary channel for depolarization measurements, where the most relevant elements are labelled.

The aerosol depolarization ratio measurement requires the comparison of the signals recovered by two channels in the system: one proportional to the total power and another proportional to the cross-polar component of the collected light [38,39]. These two channels operate at 532 nm. The depolarization auxiliary channel [19,24,27] is shown in Figure 2.

**Figure 3:**
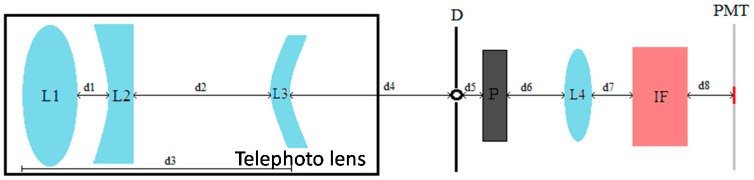
Depolarization channel optical configuration; L1 to L3 are the lenses included in the telephoto lens; L4 works as an eye-piece lens that produces an image of the telephoto lens input aperture on the PMT active surface; P is a polarizing analyzer; IF is an interference filter centered at 532 nm; distances d4 to d8 are listed in Table 1.

The depolarization channel uses a separate telescope (a 70-mm aperture, 300-mm focal distance TAIR-3S telephoto lens, BelOMO, Minsk, Belarus). The rest of the optical arrangement, sketched in Figure 3, includes a field-of-view stop iris (D) and a polarization analyzer (P) in the focal plane of the telephoto lens, and an eye-piece lens (L4) and an interference filter (IF). The polarization analyzer consists on a linear polarizer mounted on the goniometric mount that can be seen in Figure 2.

The different parameter values are indicated in Table 1. Some of the parameters provided in Table 1 have been experimentally determined and adjusted for an optimal performance of the depolarization channel. Every component (except for the telephoto lens) has a diameter of 2.54 mm. The distances between the lenses included in the telephoto lens (d1 to d3) are not provided by the manufacturer, while distance d4 has been estimated as a function of its overall performance.

The collected light is detected by the active surface of a Licel^®^ R9880U photo-multiplier module [40] (PMT in Figure 3), which feeds the detected signal to a dedicated Licel^®^ transient recorder.

The iris is located at the focal plane of the telephoto lens and limits the field of view to a theoretical value of 3.33 mrad (reduced partially due to construction compromises, as it will be pointed out later), which is essential to limit the amount of background diffuse light that reaches the PMT active surface, as it is the reduced value of the spectral width of the interference filter; the eye-piece produces an image of the telephoto lens input aperture onto the PMT active surface, spreading the collected light onto its surface.

**Figure 4:**
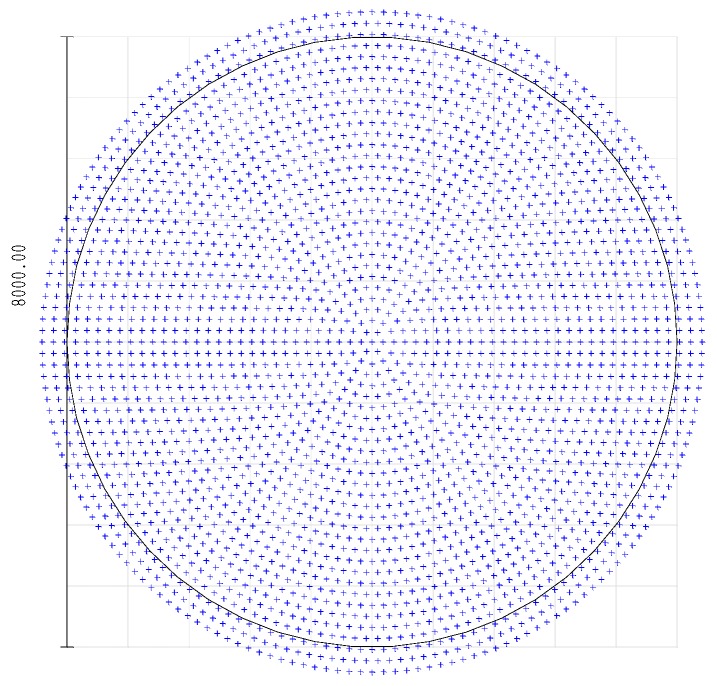
Spot diagram of the distribution of the collected rays, parallel to the optical axis, over the 8-mm diameter active surface of the photo-multiplier detector tube calculated with ZEMAX^®^ software.

A ray-optics simulation of the receiver has been performed, using the ZEMAX^®^ software (Zemax LLC, Kirkland, WA, USA). Figure 4 plots the axial ray distribution over the plane of the active surface of the photo-multiplier tube. The plot shows the impinging points of the rays parallel to the optical axis. The PMT active surface has an 8-mm diameter. According to the optical analysis all the traced rays lay within an 8.6-mm diameter, which leads to an estimated 16% overspill.

**Figure 5:**
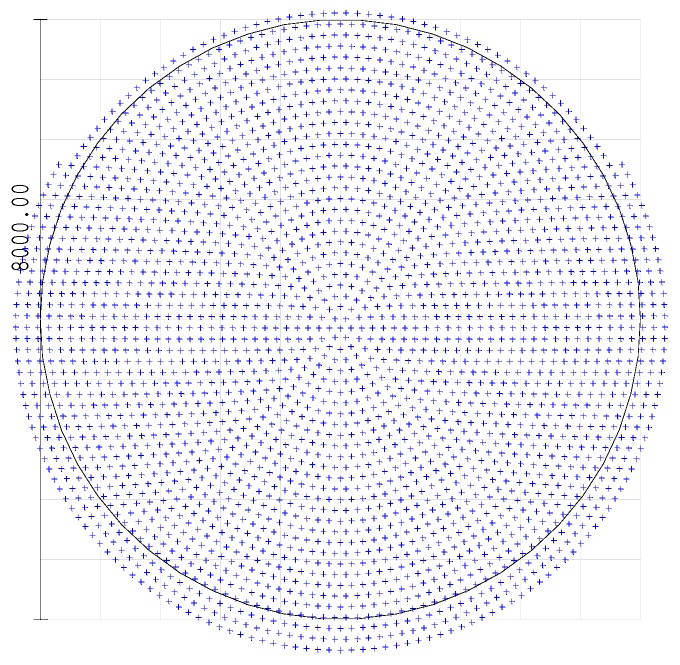
Spot diagram of the distribution of the collected extreme rays, entering the optical system with an angle equal to half the effective field of view (0.09°, over the 8-mm diameter active surface of the photo-multiplier detector tube calculated with ZEMAX^®^ software. This diagram shows that the centroid of the collected rays is displaced by approximately 130 µm in the vertical (negative sense) direction, with respect to Figure 4.

Figure 5 shows the ray distribution of those entering the telephoto lens with the maximum angle, according to the effective field of view which, according to the simulation has been reduced to 0.09° (equal to a maximum effective field of view of the optical system of approximately 3.14 mrad). For this ray distribution the centroid of the collected rays is displaced approximately 130 µm in the vertical direction, while the maximum deviation from the center of the PMT active surface is 4.41 mm, which would lead to a maximum overspill of 22% approximately. The differences with the ray distribution of Figure 4 are attributable to the non-idealities of the optical components included in the subsystem. It is likely that the choice of a shorter focal length lens for lens L4 would avoid this overspill. Some mechanical constraints prevent us currently from shortening this focal length.

**Figure 6:**
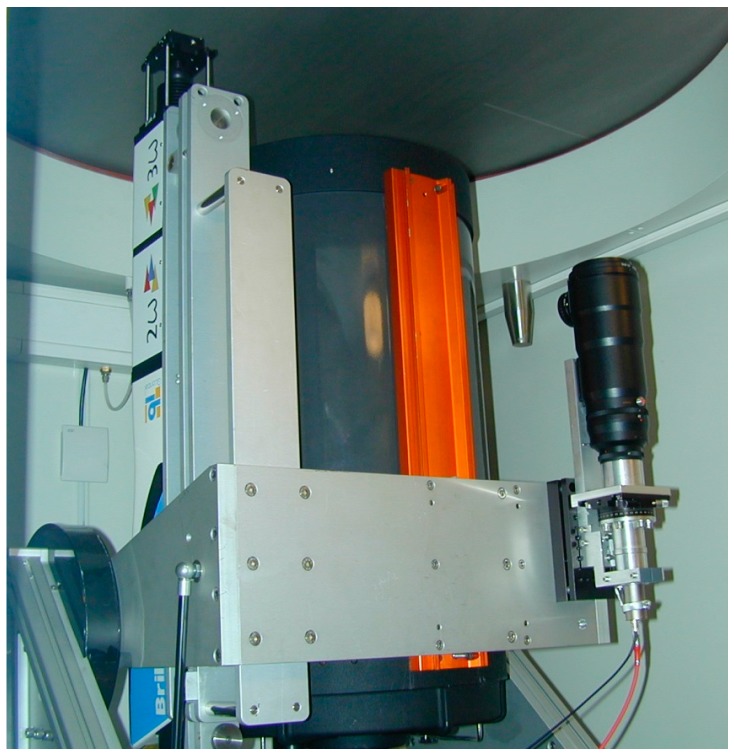
Complete view of the UPC lidar system: the laser on the left (including 2nd and 3rd harmonic generators), the main telescope in the middle and the depolarization auxiliary channel on the right.

The telephoto lens axis is approximately 40 cm from the laser beams (refer to the laser description above), which affects to the partial overlap at short distances as indicated earlier in the text. Figure 6 shows the complete system in our laboratory. The lidar is pointed vertically; whenever it is not been used, a motorized hatch protects the instrument.

The nominal position of the polarization analyzer is 90° from the transmitted beam polarization plane. In this way the channel is sensitive to the cross-polarized fraction of the light backscattered by the atmosphere.

## 3. Theory of Operation

The lidar measures along a vertical axis, so in every expression the distance R to the lidar is equivalent to the height over the system.

The voltage signal obtained at the total-power PMT output can be written as:

(1) $$S_{Tot}\left(R\right)=V_{Tot}\left(R\right)\cdot P_{Tot}\left(R\right)$$

where: *V_Tot_*(*R*) is the total-power 532-nm channel responsivity, as a function of the distance to the lidar system R, including the effect of the partial overlap (see Section 2) and *P_Tot_*(*R*) is the backscattered light power collected by the main telescope @532 nm.

The voltage signal obtained at the depolarization channel PMT output can be calculated:

(2) $$S_{Dep}\left(90{}^{\circ},R\right)=V_{Dep}\left(R\right)\cdot P_{⊥}\left(R\right)$$

where: *V_Dep_*(*R*) is the depolarization channel responsivity, as a function of the distance to the lidar system *R*, including the effect of the partial overlap, and *P*_⊥_(*R*) is the cross-polar fraction power of the depolarized backscattered light, function of *R*.

The depolarization channel system function is defined as:

(3) $$V^{*}\left(R\right)=\frac{V_{Dep}\left(R\right)}{V_{Tot}\left(R\right)}$$

While it is extremely difficult to determine *V_Dep_*(*R*) and *V_Tot_*(*R*) separately, it is possible to determine V*(*R*) by means of a calibration process that compares the output signals of the total-power channel and the cross-polar channel, when the polarization analyzer of the latter is set successively at + and −45° from its nominal position [22]:

(4) $$V^{*}\left(R\right)=2\sqrt{\frac{S_{Dep}\left(90{}^{\circ}-45{}^{\circ},R\right)}{S_{Tot}\left(R\right)}\times \frac{S_{Dep}\left(90{}^{\circ}+45{}^{\circ},R\right)}{S_{Tot}\left(R\right)}}$$

The factor 2 takes into account that, at the calibration positions, the auxiliary channel is detecting half of the total backscattered power.

The volume depolarization is defined as [22]:

(5) $$\delta^{V}\left(R\right)=\frac{P_{⊥}\left(R\right)}{P_{\|}\left(R\right)}$$

Accepting that *P_Tot_*(*R*) = *P*_‖_(*R*) + *P*_⊥_(*R*), and operating with the previous results, the volume depolarization can be calculated [36] as:

(6) $$\delta^{V}\left(R\right)=\frac{\delta^{*}\left(90{}^{\circ},R\right)}{V^{*}\left(R\right)-\delta^{*}\left(90{}^{\circ},R\right)}$$

where:

(7) $$\delta^{*}\left(90{}^{\circ},R\right)=\frac{S_{Dep}\left(90{}^{\circ},R\right)}{S_{TOT}\left(R\right)}$$

Finally, the particle depolarization ratio can be computed by combining the volume ratio with the molecular and aerosol backscattering profiles [22]:

(8) $$\delta^{p}\left(R\right)=\frac{\left[1+\delta^{m}\right]\cdot \delta^{V}\left(R\right)\cdot \rho \left(R\right)-\left[1+\delta^{V}\left(R\right)\right]\cdot \delta^{m}}{\left[1+\delta^{m}\right]\cdot \rho \left(R\right)-\left[1+\delta^{V}\left(R\right)\right]}$$

where:

(9) $$\rho \left(R\right)=\frac{\beta^{m}\left(R\right)+\beta^{p}\left(R\right)}{\beta^{m}\left(R\right)}$$

with *β^m^*(*R*) and *β^p^*(*R*) being the molecular and aerosol backscattering profiles, retrieved by means of a Klett–Fernald [41,42] or Raman [43,44] inversion performed over the signal of the total-power channel.

Finally, the molecular volume depolarization ratio:

(10) $$\delta^{m}=\frac{\beta_{⊥}^{m}}{\beta_{\|}^{m}}$$

was computed according to Behrendt and Nakamura [45] and has an approximately constant value of 3.8 × 10^−3^ for a receiver with a spectral width of 0.5 nm (see Section 2). 

The error analysis of the different magnitudes obtained in the data analysis is detailed in the Appendix A.

## 4. Calibrations

According to the previous section, the determination of the depolarization channel system function is made by means of a calibration procedure that compares the outputs of the depolarization and the total power channels [21,35,36]; during the calibration the polarization analyzer of the depolarization channel is set first at +45°, and second at −45° from the nominal (cross-polar) position. Each one of the calibrations runs for 15 minutes, which amounts to 18,000 laser pulses. The outputs of the depolarization and total power channels are divided and then a geometrical average is computed (as indicated in Equation (4)) between the system profiles obtained at the two positions; after that a zero-phase low-pass spatial filter is applied to the average, to reduce noise effects; finally, the values obtained for heights over ~10 km are discarded, also due to noise effects, considering the value obtained at 10 km for greater height values.

**Figure 7:**
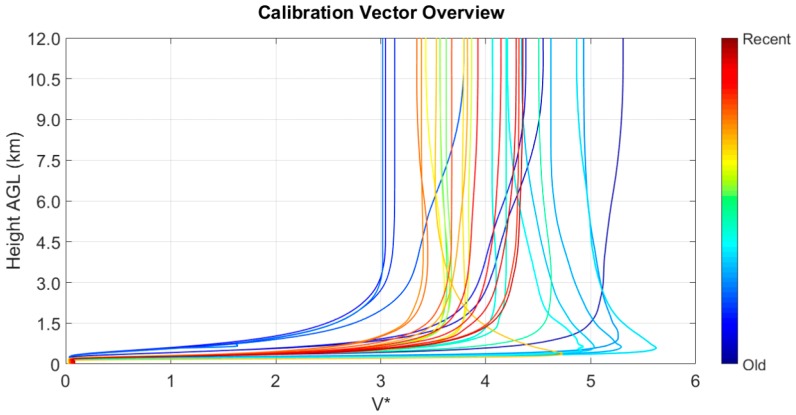
History of the calibrations of the depolarization channel system function obtained from March 2016 to June 2017. The colder colors refer to early calibrations while the warmer ones to the recent ones.

A number of calibrations have been performed since the implementation of the depolarization channel, and the history is presented in Figure 7. The color sequence shows the time evolution of the estimated system functions. As the colder colors point out, the early functions were affected by mechanical instability in the mutual alignment between the laser and the depolarization channel receiving telescope. The most recent calibrations are stabilized to a medium-height value around 4.

**Figure 8:**
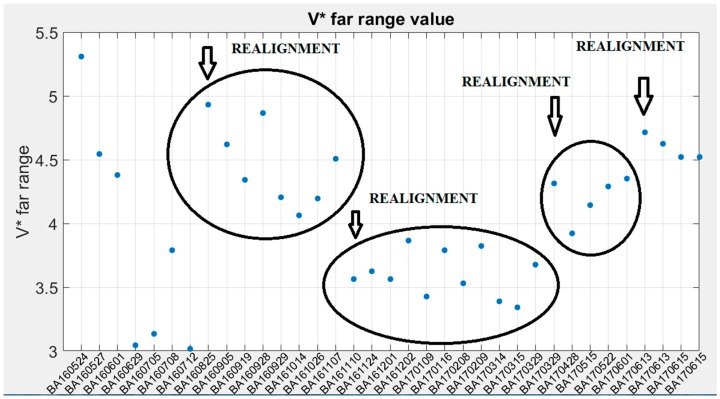
Stability of the value of the depolarization channel system function for far range; the values comprised between realignment actions are marked by closed curves.

Figure 8 shows the temporal evolution of the far range value of V* for the different calibrations presented in Figure 7 with the values obtained between realignment procedures grouped. The first group of calibrations shows a deviation exceeding 30%. After this period, an improvement in the anchorage of the receiving optics was implemented and the deviation was reduced to less than 10%, which has kept stable after successive realignment procedures. In any case, this diagram points out that different phenomena (improvements of system alignment, thermal changes, mechanical relaxation, PMT degradation…) affect V* in a way that cannot be ignored. These uncertainties make the periodic calibrations unavoidable.

The system function includes the effect of the different overlap functions [28,29,30,31,32,33,34,35] of the two channels; it also draws attention on the fact that, even though the ratio of the main telescope and the telephoto lens collecting surfaces is approximately 25, the depolarization channel optics has a higher transmission and, possibly, a PMT receiver with higher responsivity. This result also suggests that the transmission of the 532-nm total-power channel could be lower than that indicated in Section 2.

## 5. Depolarization Ratio Measurements

**Figure 9:**
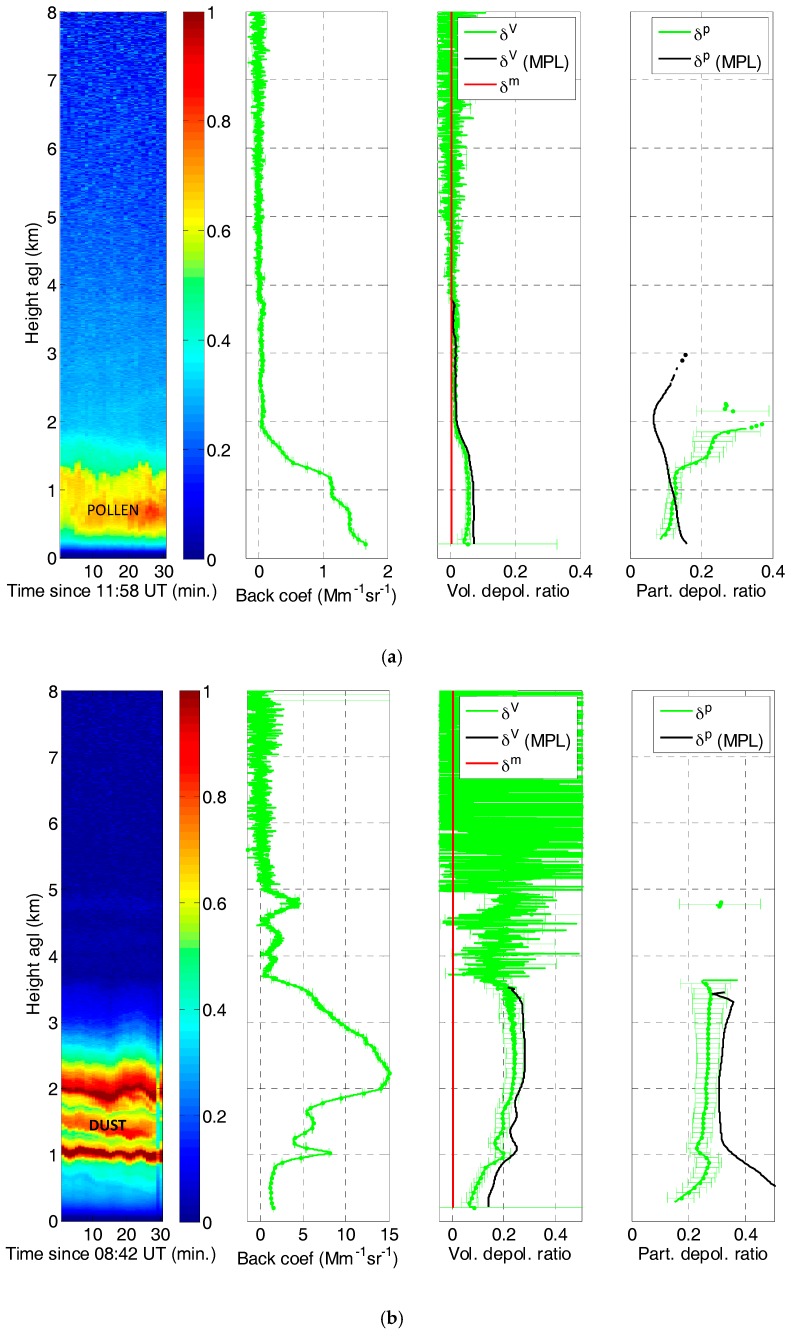

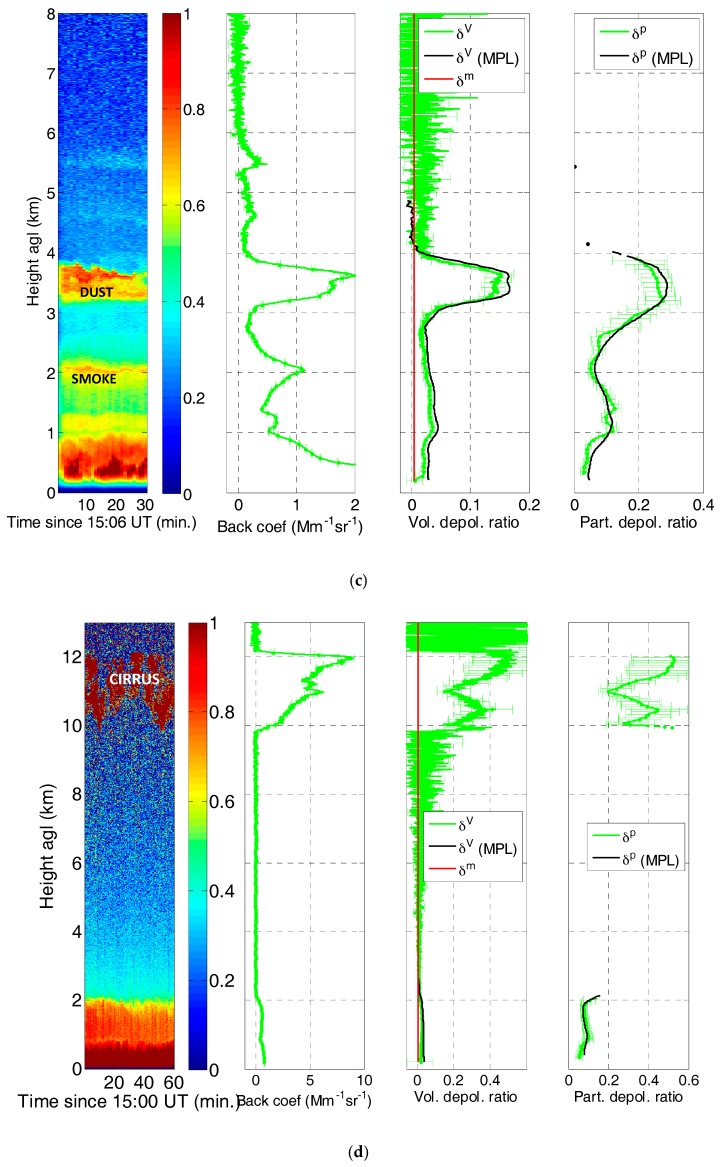

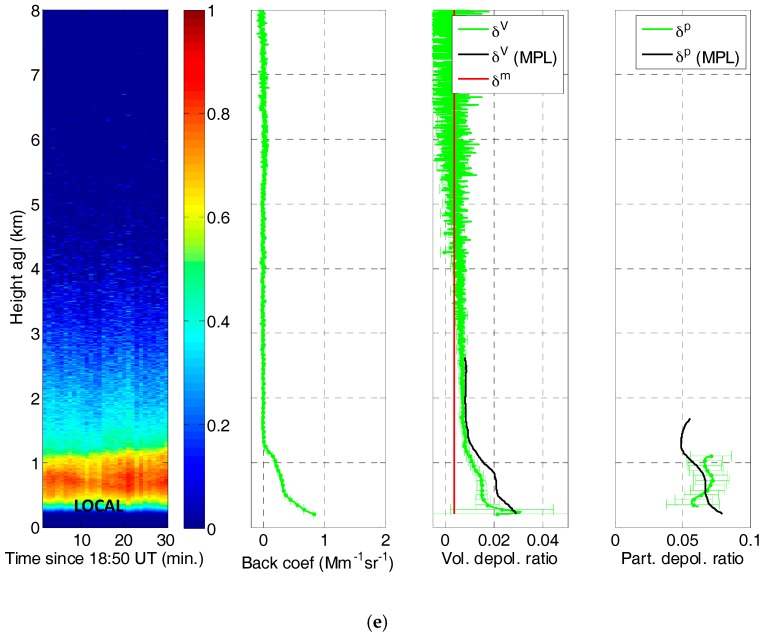
Some examples of volume and particle depolarization ratio retrievals showing (left) time-height plots of range-square corrected signals in arbitrary units, (center) particle backscatter coefficient at 532 nm, (right) volume and particle depolarization ratios at 532 nm for (**a**) pollen; (**b**) dust; (**c**) dust and fire smoke; (**d**) cirrus cloud; (**e**) local urban. The points of the particle depolarization ratio profiles for which the associated error is larger than 50% are not represented.

Some depolarization measurements are presented in Figure 9 for different aerosol loads. The volume depolarization ratio is retrieved from both total power and depolarization signals in Equation (6) and a calibration depolarization channel system function, V*(*R*). In each case the depolarization channel system function is taken from the closest (in time) calibration performed prior to the considered measurement. The particle depolarization ratio is then retrieved with Equation (8) from the volume depolarization ratio and the particle backscatter coefficient, *β^p^* [46]. All the cases presented are daytime measurements, so no Raman inversion has been performed, and thus *β^p^* has been retrieved with the Klett–Fernald [41,42] method and a constant lidar ratio of 50 srad, except in the cirrus cloud case. In this case, as there is a molecular region below and above the cloud, the iterative backward-forward method [47] was applied to invert the cloud backscatter and extinction coefficients without the need to assume a lidar ratio value. Every profile of the molecule backscatter coefficient, *β^m^*, is calculated with the closest (in time) radio-sounding either at 12 or 00 UT. The error bars are calculated following the equations detailed in the Appendix A. For the sake of clarity, the points of the profiles of the particle depolarization ratio for which the error bar is larger than 50% are not represented.

Figure 9a–e shows the retrieval of volume and particle depolarization for different aerosol loads: pollen, mineral dust, fire smoke and a case of local urban aerosol, as well as a cirrus cloud case. The measurements are compared with those from a co-located SigmaSpace MPL-4B-IDS Series micro-pulse lidar (SigmaSpace Corporation, Lanham, MD, USA) [48]. 

In the case of pollen, Figure 9a, the atmospheric boundary layer (ABL) extends up to ~1.5 km. In this layer *δ^V^* is a quite constant value of ~0.055 while *δ^p^* varies slightly between 0.10 and 0.13. These values are in agreement with depolarization ratios measured with the MPL during another pollen event in Barcelona by [49] for which we found mean values of 0.06–0.10 for *δ^V^* and 0.11–0.18 for *δ^p^*, averaged over the interval from 9:00 to 17:00 UT. For the day considered here, 14 March 2017, J. Belmonte [50] counted a total pollen near-surface concentration in Barcelona of 1746 grains per cubic meter, being 90% of them Platanus, which is in the lower range of values 1082–2830 found in [49]. The differences that can be observed in the particle depolarization ratio are very likely due to the MPL sensitivity to the overlap function correction in weak aerosol loads and to the inherent error associated to low volume depolarization ratios, particularly at low heights. In the second case, Figure 9b, we obtain typical values of depolarization for mineral dust. It is taken from an outstanding desert dust intrusion over the Iberian peninsula, which produced aerosol optical depths as large as 2 [51]. Above 1 km *δ^V^* is in the range 0.17–0.24 and *δ^p^* in the range 0.23–0.28. The small differences between *δ^V^* and *δ^p^* are due to the high values of the particle backscatter coefficient (~15 Mm^−1^ sr^−1^) inside the dust layer. According to [52] the values of *δ^p^* found in our work are in the upper range of desert dust mixtures (0.14–0.28) and below the values of pure desert dust (0.30–0.35). The agreement with the MPL measurements only takes place at height over 1 km, once again probably because of the MPL sensitivity to the overlap function correction in weak aerosol loads (here below 1 km).

The example shown in Figure 9c illustrates the transport of smoke from Canadian fires to the Iberian Peninsula on 24 May 2016, at 15 UT. The smoke layers were first detected on the evening of 22 May (see Barcelona Micro Pulse Lidar quick-looks at https://mplnet.gsfc.nasa.gov/ data?v=V3&s=Barcelona&t=20160522) and lasted until the evening of 24 May. In Figure 9c the fire smoke layer can be seen at ~2 km, under a dust layer above 3.5 km. In the fire smoke plume *δ^p^* varies in the range 0.05–0.10. Here again our findings are in agreement with the literature, in particular with [52] which presents values of *δ^p^* for pure biomass burning measured in several places around the World in the range 0.02–0.08, being values of fresh smoke slightly lower than for aged smoke. The values we present in Figure 9c fall into the interval representative of aged smoke. We extend now the illustration of particle depolarization ratios retrieved with the UPC new depolarization channel to ice particles in cirrus clouds. In this case there is a remarkable coincidence with the measurements of the co-located MPL.

Figure 9d shows a case of cirrus clouds extending between 10 and 12.2 km with a rather clean troposphere below. The application of the iterative backward-forward method [47] gives a mean cirrus lidar ratio of 19 srad and a cirrus optical depth of 0.19. The cirrus cloud is quite heterogeneous in time and vertical range during measurement (which lasted 60 min), resulting in a large variability of the particle depolarization ratio which varies between 0.20 and 0.52, being the mean value 0.39 ± 0.11. This high value of *δ^p^* is in agreement with former studies such as [53], which found values in the range 0.30–0.45 for cirrus clouds at ~9.5–11.5 km height observed in north-central Oklahoma. It must be pointed out that our MPL cannot detect aerosol or cloud structures located at such high altitudes during daytime given its limited working range.

Finally to give an idea of the particle depolarization ratio in background conditions in Barcelona, i.e., when the aerosol is from local urban origin and probably mixed with marine particles, a case without mesoscale transport as this is presented in Figure 9e. The atmospheric boundary layer is developed up to 1.25 km. *δ^p^* is nearly constant and its mean value is 0.066 ± 0.005. If we compare this value to the collection of depolarization ratios of [52] measured around the globe for anthropogenic pollution (0.06–0.10) and marine aerosols in different relative humidity conditions (0.01–0.10) we find a good agreement. However, at this point, from the depolarization ratio alone it does not seem possible to distinguish the fractions of anthropogenic pollution and of marine particles. In this case the measurements match well with those from the MPL.

## 6. Conclusions

A new depolarization sensing subsystem has been implemented to a 6-channel elastic/Raman lidar. The architecture is based on a dedicated sub-telescope (a telephoto lens). The theory of operation has been presented, including the calibration procedure. Measurements performed during different aerosol load situations are presented: pollen, dust, fire smoke, cirrus cloud and local urban conditions. Comparisons of the volume depolarization with a co-located single-wavelength, polarization-sensitive elastic MPL system show a good agreement between both systems and demonstrate the reliability of the new depolarization channel of the UPC multi-wavelength lidar.

## Figures and Tables

**Table 1:** Parameters of the different optical elements of the depolarization channel.

Parameter	Value
d4	138.9 mm (estimated)
d5	1 mm
d6	39.4 mm
d7	5 mm
d8	23 mm
Telephoto lens focal length	300 mm
Eye-piece lens focal length	38 mm
Field-of-view stop iris diameter	1 mm
**Interference filter**	BARR 532-0.5 nm (custom made)
Center wavelength	531.9 nm
Spectral width	0.5 nm
Thickness	11 mm

## Appendix A. Error Estimation

The error analysis presented in this section is based in the well-known technique of the error propagation [54,55]. If we have a function *y* that depends on *n* uncorrelated variables *x_i_*:

(A1) $$y=f=(x_{1},x_{2},\ldots ,x_{n})$$

which are known with a standard deviation $\Delta x_{i}$.

The most reliable value of y can be computed as:

(A2) $$\bar{y}=f=({\bar{x}}_{1},{\bar{x}}_{2},\ldots ,{\bar{x}}_{n})$$

With a standard deviation that can be computed as [43,44]:

(A3) $$\Delta y=\sqrt{\sum_{i=1}^{n}{\left({\frac{\partial f}{\partial x_{i}}|}_{{\bar{x}}_{i}}\right)}^{2}\;{\left(\Delta x_{i}\right)}^{2}}$$

where ${\frac{\partial f}{\partial x_{i}}|}_{{\bar{x}}_{i}}$ is the partial derivative of function *f* with respect to variable *x_i_*, evaluated in the average value $\bar{x_{i}}$.

According to this method, the following standard deviations can be obtained for the observable $\delta^{*}\left(90{}^{\circ},R\right)$, defined in Equation (7):

(A4) $$\Delta \delta^{*}\left(90{}^{\circ},R\right)=\sqrt{{\left(\frac{1}{\bar{S_{TOT}\left(R\right)}}\right)}^{2}{\left(\Delta S_{Dep}\left(90{}^{\circ},R\right)\right)}^{2}+{\left(\frac{\bar{S_{Dep}\left(90{}^{\circ},R\right)}}{{\bar{S_{TOT}\left(R\right)}}^{2}}\right)}^{2}{\left(\Delta S_{TOT}\left(R\right)\right)}^{2}}$$

where:

$\bar{S_{TOT}\left(R\right)}$ is the average value of the signal detected by the total power channel, as a function of range,
$\bar{S_{Dep}\left(90{}^{\circ},R\right)}$ is the average value of the signal detected by the depolarization channel (with the polarizer oriented 90° from the transmitted beam polarization,
$\Delta S_{TOT}\left(R\right)$ is the standard deviation of the signal detected by the total power channel.
$\Delta S_{Dep}\left(90{}^{\circ},R\right)$ is the standard deviation of the signal detected by the depolarization channel.

According to the calibration method, the uncertainty associated to the estimation of the depolarization channel system function is reduced by the signal smoothing that is performed. According to the different calibrations presented in Figure 7, an absolute error around $\left|\Delta V^{*}\left(R\right)\right|=1$ will be considered in the computation of the error of the volume depolarization ratio, defined in Equation (6):

(A5) $$\Delta \delta^{V}\left(R\right)=\sqrt{{\left(\frac{V^{*}\left(R\right)}{V^{*}\left(R\right)-\delta^{*}\left(90{}^{\circ},R\right)}\right)}^{2}{\left(\Delta \delta^{*}\left(90{}^{\circ},R\right)\right)}^{2}+{\left(\frac{\delta^{*}\left(90{}^{\circ},R\right)}{V^{*}\left(R\right)-\delta^{*}\left(90{}^{\circ},R\right)}\right)}^{2}{\left(V^{*}\left(R\right)\right)}^{2}}$$

The computation of the error of the backscatter ratio, defined in Equation (9), considers only the random variations of the retrieved particle backscatter [30,31,32,33]:

(A6) $$\Delta \rho \left(R\right)=\sqrt{{\left(\frac{1}{\beta^{m}\left(R\right)}\right)}^{2}\cdot {\left(\Delta \beta^{p}\left(R\right)\right)}^{2}}$$

Finally, for the computation of the error of the particle depolarization ratio, defined in Equation (8), we will simplify the expression by defining:

(A7) $$\begin{array}{l} Num\left(R\right)=\left[1+\delta^{m}\right]\cdot \delta^{V}\left(R\right)\cdot \rho \left(R\right)-\left[1+\delta^{V}\left(R\right)\right]\cdot \delta^{m}\\ Den\left(R\right)=\left[1+\delta^{m}\right]\cdot \rho \left(R\right)-\left[1+\delta^{V}\left(R\right)\right] \end{array}$$

And then writing:

(A8) $$\begin{array}{cc} {\left(\Delta \delta^{p}\left(R\right)\right)}^{2} & ={\left(\frac{\left[\left(1+\delta^{m}\right)\cdot \rho \left(R\right)-\delta^{m}\right]\cdot Den\left(R\right)+Num\left(R\right)}{{\left(Den\left(R\right)\right)}^{2}}\right)}^{2}\cdot {\left(\Delta \delta^{v}\left(R\right)\right)}^{2}+\\ & +{\left(\frac{\left(1+\delta^{m}\right)\cdot \delta^{v}\left(R\right)\cdot Den\left(R\right)-\left(1+\delta^{m}\right)\cdot Num\left(R\right)}{{\left(Den\left(R\right)\right)}^{2}}\right)}^{2}\cdot \left(\Delta \rho \left(R\right)\right) \end{array}$$
